# Dietary inflammatory index and risk of gallstone disease in Iranian women: a case-control study

**DOI:** 10.1186/s12876-023-02943-9

**Published:** 2023-09-14

**Authors:** Moloud Ghorbani, Azita Hekmatdoost, Zahra Darabi, Amir Sadeghi, Zahra Yari

**Affiliations:** 1 Department of Community Nutrition, Faculty of Nutrition and Food Sciences, Tabriz University of Medical Sciences, Tabriz, Iran; 2 Clinical Nutrition and Dietetics Department, Faculty of Nutrition Sciences and Food Technology, National Nutrition and Food Technology Research Institute, Shahid Beheshti University of Medical Sciences Tehran, Tehran, Iran; 3 Department of Nutrition, School of Public Health, Shahid Sadoughi University of Medical Sciences, Yazd, Iran; 4 Nutrition and Food Security Research Center, Shahid Sadoughi University of Medical Sciences, Yazd, Iran; 5 Department of Adult Gastroenterology and Hepatology, School of Medicine, Shahid Beheshti University of Medical Sciences, Tehran, Iran; 6 Department of Nutrition Research, National Nutrition and Food Technology Research Institute, Faculty of Nutrition Sciences & Food Technology, Shahid Beheshti University of Medical Sciences, Tehran, Iran

**Keywords:** Gallstone disease, Dietary inflammatory index, Inflammation

## Abstract

**Background:**

Considering inflammation as a primary occurrence in gallstone formation, this study aimed to determine the relation between serum biomarkers of inflammation and oxidative stress, and dietary Inflammatory Index (DII) score with the risk of gallstone disease (GD) among Iranian women.

**Materials and methods:**

Present BMI-matched case-control study was performed among 75 women with GD and 75 healthy controls. Biochemical parameters were measured by standard laboratory methods. A validated food frequency questionnaire (FFQ) was used to assess the usual intake of participants. DII score was calculated for all participants. The linear and logistic regression were used to examine the association of DII with serum inflammatory biomarkers and the odds ratio of GD, respectively.

**Results:**

The mean serum levels of high-sensitivity C-reactive protein (hs-CRP) and Malondialdehyde (MDA) were significantly (*P* < 0.001) higher in GD patients compared to control subjects. Women in the highest tertile of DII compared to the lowest tertile had lower intake of macronutrients, minerals, vitamins garlic, onion, pepper and fiber. Moreover, the odd of GD was significantly higher in the third tertile of the DII versus the first tertile after adjustment of potential confounders (OR: 17.47; 95% CI: 4.64–65.72). Also, a positive and significant relationship was found between the serum level of inflammatory biomarkers with the risk of GD and the inflammatory score of the diet (P < 0.001).

**Conclusion:**

Our data indicate that higher DII score, and serum inflammatory and oxidative stress biomarkers are related to higher risk of GD in Iranian women.

## Introduction

Gallstone disease (GD) is one of the most common disorders associated with gastrointestinal tract [1]. Formation of gallstones has been attributed to cholesterol-supersaturated bile, nucleation of cholesterol crystals and altered gallbladder emptying [2]. GD Complications such as cholecystitis, cholangitis and pancreatitis lead to hospital admission and surgical interventions that represent a major economic burden on healthcare resources [3, 4]. Recent studies have shown that the prevalence of gallstones is between 15 and 20% in Western countries [5]. In the US about 35% of GD cases result in cholecystectomy and more than 750,000 cases of cholecystectomy occur annually [6]. The prevalence of gallstone disease in Iran was reported 6.3% by a study conducted in 2007 [7].

GD is a multifactorial disorder and probably both genetic and environmental factors are involved in its pathogenesis. Female sex, older age, higher body mass index (BMI), rapid weight loss, pregnancy, liver cirrhosis, hemolytic anemia, use of certain therapeutic agents (Estrogen-treatment when used for anticonception or hormone-replacement and fibrates used for the treatment of dyslipidemia), hyperlipidemia and diabetes mellitus increase the risk of GD [1, 8–10]. Diet has long been considered a modifiable risk factor for GD [11]. Previous studies have reported that the risk of GD is positively associated with meat consumption, high intake of energy, fats and saturated fatty acids, but is inversely related to the consumption of vegetables and fiber [12]. Regarding diet as an important ecological factor, the evidence in nutrition epidemiology suggests that pattern analysis compared with a dietary component is the most functional way to assess the effects of whole diet on health or disease [11].

It has been demonstrated that inflammation is a primary occurrence in gallstone formation [13]. The studies have reported that there is a significant association between circulating inflammatory biomarkers and the risk of GD [13, 14]. Considering to anti-inflammatory/pro-inflammatory potential capacity of specific foods and dietary patterns, they could be related to GD [15].

The Dietary Inflammatory Index (DII) is a population-based diet quality index which derived from literature; include the positive or negative outcomes of 45 various dietary factors on serum levels of six inflammatory biomarkers. A higher score of DII indicates a higher inflammatory potential of the diet [16]. Although results from a review study show that high DII might be related to an elevated risk of cardiovascular disease, colorectal cancer and all-cause mortality [17], but there is little information about the relationship between DII and the odds of gallstone disease. Since the studies in this field are limited, therefore this study aimed to determine the association between DII score and the odds of GD in women.

## Materials and methods

### Participants

This BMI-matched case-control study was performed in Research Institute for Gastroenterology and Liver Diseases of Shahid Beheshti University of Medical Science in Tehran, Iran from October 2020 to March 2021. This study was approved by the Ethical Committee of the Faculty of Nutrition and Food Sciences, Tabriz University of Medical Sciences, Tabriz, Iran (research ethic number: IR.TBZMED.REC.1398.1202). At the beginning of study, all participants completed an informed written consent form. All methods were carried out in accordance with the Declaration of Helsinki and Good Clinical Practice guidelines.

Subjects included 75 new cases of women (aged 30–65 years) with GD or common bile duct stone (CBD), which was confirmed by ultrasonography. Furthermore, the control group consisted of 75 healthy women (aged 30–65 years) from the companions of the patients. Sample size using PASS software and according to the study of Hayat et al. [18] (SD values for triglyceride variable), considering the test power of 80%, 68 people in each group and finally by 10% drop, 75 people in each group was calculated.

Main inclusion criteria were women aged 30–65 years, newly diagnosed with GD, not taking medicines to control blood sugar or lipids, or Ursodeoxycholic Acid, lack of history of diabetes and cardiovascular disease, not following certain diets, and readiness to participation. Criteria for exclusion from the study consisted of: pregnancy or lactation, consumption of vitamin and mineral supplements and taking weight loss medications.

### Assessment of dietary intake

A validated 125-item food frequency questionnaire (FFQ) [19] was used to assess the usual intake of foods and beverages in the past year. These food items in FFQ had been classified into 7 food groups and the participant’s response to the frequency of consumption for each food item was ranged from “never or less than once a month” to “12 or more times per day”. Using household measures, portion sizes of consumed foods were converted to grams [20]. The Nutritionist IV software (First Databank, San Bruno, CA, USA) that was updated for Iranian foods was used to evaluate the recorded foods. All FFQs were completed by a trained dietitian.

### Calculation of Dietary Inflammatory index (DII)

The Dietary Inflammatory Index (DII) was computed according to the updated approach of Shivappa et al. [16, 21]. DII was established based on the effect of 45 nutritional parameters (including macronutrients, micronutrients, specific food, and bioactive ingredients) on inflammatory indices. Each dietary parameters were scored based on how their affect inflammation considering to six inflammatory indicators (Interleukin (IL) − 1β, IL-4, IL-6, IL-10, tumor necrosis factor (TNF) and C reactive protein (CRP)); foods with potential inflammatory effect (+ 1 point) or anti-inflammatory effect (-1point) [16, 21, 22]. In the present study, the following 30 food items were available to calculate the DII score: energy, carbohydrate, protein, total fat, cholesterol, vitamin B12, vitamin B6, β-Carotene, caffeine, fiber, folic acid, garlic, Fe, Mg, MUFA, niacin, n-3 Fatty acids, n-6 Fatty acids, onion, PUFA, riboflavin, saturated fat, Se, thiamin, vitamin A, vitamin C, vitamin D, vitamin E, Zn, pepper. Diet information were compared to a global database that supplied the estimated mean and standard deviation (SD) for daily intake of each DII parameter from 11 countries. Z-score is obtained via subtracting a standard global was from the reported intake and divided by its SD. Then Z-scores were converted to percentiles. The percentiles for each food parameter were multiplied by 2 and 1 was subtracted. Next, the obtained number for each parameter was multiplied by the inflammatory effect score of that parameter. Finally, the DII scores of each parameter were summed to gain a total DII score for any participant. A higher DII score refer to a greater consumption of pro-inflammatory foods, while a lower score indicates a greater intake of anti-inflammatory foods [23].

### Assessment of biomarkers

After 12 h of fasting, venous blood samples were collected from all participants. All measurements were performed on the extracted serum samples that stored at − 80 °C. Serum hs-CRP was measured by immunoturbidimetry method (Pars Azmoon Iran). Measurement of serum MDA was performed by spectrophotometric method based on reaction with thiobarbituric acid (TBA). All biochemical parameters were assessed in one laboratory by a trained laboratory expert.

### Assessment of other variables

Physical activity (PA) was also determined with the short form of the international physical activity questionnaire (IPAQ) and expressed as physical activity levels [24]. In addition, anthropometric measurements including body weight and height were collected after an overnight fasting by a trained dietician, while subjects were wearing light indoor clothing without shoes. Height was measured to the nearest 0.1 cm with a measuring tape. Weight was recorded to the nearest 0.1 kg with a Butcher scale (Seca, Hamburg, Germany). Body mass index (BMI) was calculated dividing weight (kg) by the square of height (m2) [25].

### Statistical methods

Normality of data was checked by Kolmogorov-Smirnov test. All variables had normal distribution. Quantitative variables were reported as mean ± standard deviation and categorical variables were expressed as percentage. To compare continuous and categorical variables between two groups, independent sample t-test and chi-square test were used, respectively. To compare the mean of quantitative and qualitative variables across DII tertiles, the one-way analysis of variance and chi-square tests were conducted, respectively. In order to ascertain the odds of GD in each tertile of DII, the logistic regressions were applied in crude and multivariable-adjusted model. Confounding variables adjustment including age, BMI, energy intake, IPAC (level), smoking, MDA and hs-CRP were performed in the adjusted model. Linear regression was employed to assess the association between GD with the serum levels of hs-CRP and MDA. All statistical analyses were performed using the Statistical Package for the Social Sciences (SPSS) software version 25.0 (SPSS Inc., Chicago, IL, USA). Two-sided P-values < 0.05 were considered significant.

## Results

General characteristics of participants are shown in Table 1. There were no significant differences in terms of BMI, PA and smoking, between case and control groups; whereas the mean age was significantly higher among women with GD. The mean serum levels of hs-CRP (*P* < 0.001) and MDA (*P* < 0.001), were significantly higher in GD patient compared to control subjects.

**Table 1:** Characteristics of the study participants across tertiles of DII also base on case and control groups

Variables	Participants				Dietary Inflammatory Index			
	**Case** **n = 74**		**Control** **n = 75**	***P*** ^**a**^	**T1** **n = 49**	**T2** **n = 50**	**T3** **n = 50**	***P*** ^**b**^
General characteristics								
Age (year)	51.47 ± 12.54		44.16 ± 9.03	**< 0.001**	45.40 ± 10.62	47.68 ± 11.42	50.24 ± 12.04	0.11
Weight (kg)	69.29 ± 11.16		72.74 ± 9.82	**0.04**	74.85 ± 9.88	70.20 ± 7.73	68.12 ± 12.69	**0.005**
Height (cm)	160.16 ± 5.64		161.41 ± 7.43	0.25	161.06 ± 7.37	161.10 ± 6.53	160.22 ± 5.97	0.757
BMI (kg/m^2^)	27.06 ± 4.61		27.90 ± 3.11	0.19	28.86 ± 3.30	27.06 ± 2.75	26.55 ± 5.06	**0.008**
Smoking				0.37				0.20
Yes		2 (2.7%)	0 (0.00)		0	2(4%)	1(2%)	
No		72 (97.3%)	75 (100)		49 (100%)	48 (96%)	49(98%)	
IPAC (level)				0.57				0.16
Light		59 (79.7%)	58 (77.3%)		34 (69.4)	38 (76%)	45 (90%)	
Medium		10 (13.5%)	14 (18.7%)		11 (22.4)	9 (18%)	4 (8%)	
Severe		5 (6.8%)	3 (4%)		4 (8.2)	3 (6%)	1 (2%)	
Clinical markers								
hs-CRP (mg/dL)	14.91 ± 9.84		5.35 ± 3.51	**< 0.001**	8.11 ± 6.87	9.54 ± 8.11	12.61 ± 10.46	**0.03**
MDA (nmol/mL)	3.97 ± 3.07		1.66 ± 0.40	**< 0.001**	2.28 ± 1.69	2.11 ± 1.27	4.04 ± 3.41	**< 0.001**

BMI: Body mass index; hs-CRP: high-sensitivity C-Reactive Protein; MDA: Malondialdehyde; DII: Dietary Inflammatory Index

Values are means ± SD and number (prevalence percent) for quantitative and qualitative variables, respectively.

^a^ Obtained from Independent t-test for quantitative variables and Chi-squared test for qualitative variables

^b^ Obtained from one way ANOVA for continuous variables and Chi-squared test for categorical variables

DII components of study population in tertiles of DII are represented in Table 2. Women in the highest tertile of DII compared to lowest tertile had significantly lower intake of macronutrients, MUFA, PUFA, n-3 fatty acid, n-6 fatty acid, minerals, vitamins, fiber, onion and pepper.

**Table 2:** DII components of study participants by tertiles of DII

Variables	Dietary Inflammatory index			*P* ^*^
DII components	**T1** **n = 49**	**T2** **n = 50**	**T3** **n = 50**	
Macronutrients				
Energy (Kcal)	3194.93 ± 903.47	2341.69 ± 498.61	1835.54 ± 488.49	**< 0.001**
Protein (g)	94.14 ± 27.67	67.86 ± 13.41	49.82 ± 14.24	**< 0.001**
Carbohydrate (g)	405.80 ± 107.911	290.88 ± 64.09	225.36 ± 60.65	**< 0.001**
Fat (g)	142.36 ± 60.29	107.53 ± 41.37	86.83 ± 37.49	**< 0.001**
Fatty acid subtypes				
Cholesterol) mg (	277.19 ± 103.27	190.38 ± 64.80	152.94 ± 88.20	**< 0.001**
SFA (g)	34.72 ± 13.49	26.31 ± 7.53	22.43 ± 8.15	**< 0.001**
MUFA (g)	48.71 ± 20.94	38.23 ± 14.04	31.08 ± 14.67	**< 0.001**
PUFA (g)	35.00 ± 17.49	27.42 ± 12.63	22.09 ± 13.30	**< 0.001**
n-3 Fatty acid (g)	18.02 ± 15.23	13.93 ± 12.69	9.30 ± 8.85	**0.003**
n-6 Fatty acid (g)	31.61 ± 16.96	24.86 ± 12.19	20.08 ± 12.68	**< 0.001**
Minerals				
Iron (mg)	19.86 ± 5.52	14.07 ± 3.22	10.14 ± 2.98	**< 0.001**
Magnesium (mg)	458.61 ± 88.25	328.68 ± 65.67	242.82 ± 70.24	**< 0.001**
Zinc (mg)	14.36 ± 4.44	9.92 ± 2.00	7.29 ± 2.29	**< 0.001**
Selenium (mg)	119.98 ± 41.68	79.64 ± 22.99	61.48 ± 22.82	**< 0.001**
Vitamins				
Vitamin A (mg)	653.71 ± 257.56	415.32 ± 187.26	310.55 ± 109.41	**< 0.001**
Betacaroten (mg)	3683.39 ± 1370.99	2600.96 ± 1355.33	1776.31 ± 910.91	**< 0.001**
Vitamin C (mg)	229.96 ± 95.77	164.29 ± 78.74	105.84 ± 62.14	**< 0.001**
Vitamin D (µg)	1.96 ± 1.33	1.16 ± 1.24	0.91 ± 0.86	**< 0.001**
Vitamin E (mg)	36.08 ± 23.55	28.18 ± 18.69	20.27 ± 14.09	**< 0.001**
Thiamin (mg)	2.17 ± 0.74	1.58 ± 0.36	1.20 ± 0.34	**< 0.001**
Riboflavin (mg)	2.10 ± 0.65	1.55 ± 0.36	1.15 ± 0.58	**< 0.001**
Niacin (mg)	26.10 ± 8.92	18.56 ± 4.21	13.51 ± 4.12	**< 0.001**
Folate (mg)	656.99 ± 192.59	495.47 ± 96.20	361.09 ± 92.63	**< 0.001**
Vitamin B6 (mg)	2.37 ± 0.63	1.75 ± 0.34	1.26 ± 0.36	**< 0.001**
Vitamin B_12_ (mg)	5.38 ± 2.75	3.62 ± 1.52	2.57 ± 1.58	**< 0.001**
Other nutrients				
Total Fiber (g)	37.43 ± 10.12	27.43 ± 7.38	19.48 ± 7.53	**< 0.001**
Caffeine (g)	168.13 ± 115.25	156.04 ± 98.20	149.19 ± 97.87	0.060
Garlic (g)	0.81 ± 1.66	1.08 ± 2.08	1.22 ± 4.62	0.790
Onion (g)	34.66 ± 48.89	19.29 ± 23.19	7.66 ± 14.91	**< 0.001**
Pepper (g)	16.00 ± 17.49	10.90 ± 12.40	6.49 ± 10.54	**0.003**

DII: Dietary Inflammatory Index

Values are means ± SD

*Obtained from One way ANOVA

**Fig. 1:**
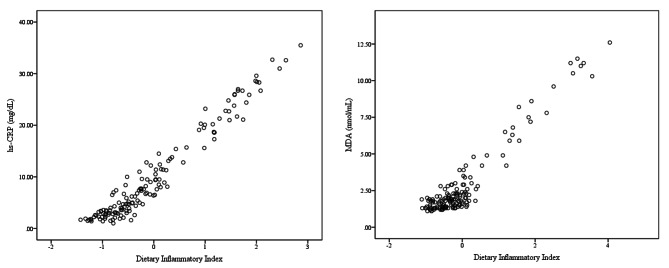
The association between DII with serum hs-CRP and MDA. P < 0.001. hs-CRP: high-sensitivity C-reactive protein; MDA: Malondialdehyde

Odds ratios of crude and adjusted models for GD across tertiles of DII are demonstrated in Table 3. There was a significant positive association between DII and odds of GD (OR: 1.08; 95% CI: 0.51–0.77) in a crude model. Moreover, the odds of GD were significantly higher in third tertile of the DII versus first tertile in a crud model. This association remained significant after adjustment of potential confounders including energy intake, BMI, PA and smoking (OR: 17.47; 95% CI: 4.64–65.72; p third tertile compare to first tertile: <0.001). The association between GD with the serum inflammatory biomarkers are indicated in Table 4 in crude and multivariable-adjusted models. The linear regression analysis showed that there is a significant positive association between GD and serum levels of hs-CRP and MDA in either crude or confounder-adjusted models (P < 0.001). The DII score was also linearly associated with serum inflammatory biomarkers (Fig. 1). The analysis revealed that DII score is associated with higher serum levels of hs-CRP and MDA (P < 0.001).

**Table 3:** Odds ratio of GD across tertiles of dietary inflammatory index

DII	OR (95% CI)			P*
	**T1**	**T2**	**T3**	
Crude Model	1.00	1.93 (0.84–4.40)	5.82 (2.45–13.85)	**< 0.001**
Model 1	1.00	3.72 (1.26–10.98)	17.47 (4.64–65.72)	**< 0.001**
Model 2	1.00	4.97 (1.14–21.55)	11.49 (1.83–71.99)	**< 0.01**

DII: Dietary Inflammatory Index

Model 1: Adjusted for Age, BMI, Energy, IPAC (level), smoking

Model 2: Adjusted for Model 1 + MDA, hs-CRP

^*^P T3 compare to T1

**Table 4:** The association between GD with the serum levels of hs-CRP and MDA

Variables	hs-CRP		MDA	
GD	β*	*P*	β*	*P*
Crude Model	0.54	**< 0.001**	0.46	**< 0.001**
Adjusted Model ^λ^	0.50	**< 0.001**	0.44	**< 0.001**

GD: Gallstone Disease

^λ^ Adjusted for Age, BMI, Energy, IPAC (level), smoking

^*^Linear Regression

## Discussion

To the best of our knowledge, this study was the first case-control study that examined the association between DII and the odds of GD among women. Our findings demonstrated that a higher score of DII can increase the odds of GD.

Several studies have investigated the role of inflammation in gallstone formation. In a case-control study by Liu et al. [14] in order to find the relationship between inflammatory proteins and GD, 13 cytokines including 10 interleukins were measured and determined that IL-6, IL-10, IL-12, and IL-13 were associated with elevated risk of GD. As in our study, the mean serum level of hs-CRP in patients was significantly higher than the control group and there was a significant increase in hs-CRP level with the increase in the DII tertile, in a recent cohort study with a more than 7-year follow-up, it has been reported that high levels of serum hs-CRP, was an independent risk factor for new-onset GD formation amongst the Chinese people [26]. Results from a cross-sectional study by Shabanzadeh et al. showed that CRP as one of the systemic inflammation agents had a positive relationship with GD [13]. Also, in line with our results, in three case-control studies by Atamer et al. and Ridha et al. and AL-Ta’ai et al. serum concentration of MDA in GD patients was significantly higher than healthy subjects [27–29].

It has been shown that a diet high in meat, animal fats and fried foods increases the risk of GD; On the other hand, a high intake of fruits, vegetables, nuts, fish, MUFA and n-3 fatty acids can play a protective role against GD [30]. Energy intake, macronutrients and micronutrients including folate, vitamin C, niacin, calcium and magnesium are also linked with GD [31, 32]. A recent case-control study demonstrated that higher adherence to a diet rich in fruits, vegetables, legumes, nuts, vegetable oils and fish, as well as a lower intake of hydrogenated fats and salt, decreased the risk of GD [33]. Also, results from a cohort study showed that higher consumption of fruits, vegetables and foods with a high fiber content can decrease the risk of GD while increasing the red meat intake, foods rich in cholesterol and saturated fat can increase the risk of GD [34]. The DII reflects the potential inflammatory effect of diets based on the consumption of inflammatory or anti-inflammatory foods [16]. Previous studies have shown a relationship between high scores of DII with cardiovascular disease, metabolic syndrome, cancer, and also neuropsychiatric disorders [35–38]. In contrary to our result, Sadri et al. in a cross-sectional study demonstrated that a proinflammatory diet is associated with decreased odds of GD [15]. The reason for this inconsistency may be related to the study’s design, as our study included new cases, while Sadri et al. examined baseline data of a cohort study.

Although it is well-established that inflammation involves in gallstone formation, the exact mechanisms are still unclear [14]. Inflammation may change multiple proteins and lipids metabolism; these alterations may cause a change in the metabolism of cholesterol and bile acids, and following that increase bile salt levels, therefore can result in gallstone formation [14]. IL-6 can be expressed by the gallbladder epithelium as well as activate the proliferation of several non-hematopoietic cells. IL-6 can also result in the penetration of inflammatory cells and increase the gallbladder wall thickness [39]. It has been recognized that there is a link between the thickness of the gallbladder wall and its motility [39, 40]. Thus, gallbladder inflammation might lead to gallbladder dysmotility. IL-12 via induce the secretion of TNF-α from T cells and natural killer cells, has direct effect on the absorption, secretion, and functions of gallbladder epithelial cells [41]. Prior studies show that adipocytes by stimulating the secretion of interleukin-6, contributed to the generation of CRP in the liver [42]. It has been illustrated that chronic inflammation of any cause might lead to calcium deposition in the gallbladder wall, which may describe the association between CRP and GD [43]. Increased production of ROS (reactive oxygen species) and toxic products resulting from lipid peroxidation such as malondialdehyde has been observed in the GD patient’s serum samples [44]. It is well-identified that, in chronic diseases like GD, the active inflammatory response is induced via neutrophilic infiltration. These neutrophils, monocytes and/or macrophages generate ROS which may result in DNA damage to the contiguous cells [45, 46]. Oxidative stress onset is a process causing the synthesis of pro inflammatory cytokines and cell adhesion molecules. Thus, oxidative stress may assist in an inflammatory response caused by GD [44].

Although this was the first case-control study that examined the relation of DII and GD among women and results were adjusted for age, BMI, energy intake, IPAC level and smoking as confounders; nevertheless, this study had some limitations. First, the sample size of the study population was small. Second, due to our study only included women, our results cannot be generalized to both genders. Furthermore, use of FFQ questionnaire for participants’ dietary assessment is inevitably subject to error. Due to the subjective nature this method, over- or under-reporting of food intake cannot not be predicted, therefore more detailed studies are required.

## Conclusion

Our results revealed that there is a significant direct association between DII and GD. More mechanism-based studies are needed to support these findings in the future.

## Data Availability

All data generated or analyzed during this study are included in this published article.
